# Supplementary material for: Analysis of chi angle distributions in free amino acids via multiplet fitting of proton scalar couplings
Source: Magn Reson (Gott). 2024 Aug 19;5(2):103–20. doi: 10.5194/mr-5-103-2024 (PMC11570886; doi:10.5194/mr-5-103-2024)
Supplement: The supplement related to this article is available online at: https://doi.org/10.5194/mr-5-103-2024-supplement. [file mr-5-103-2024-supplement.zip › Syed2024/data/fit1d_fitnmr_output.tar.gz!/fit1d/ile/500/bmse000041_simulation_1_nmredata/1H/resonances_1d.pdf]

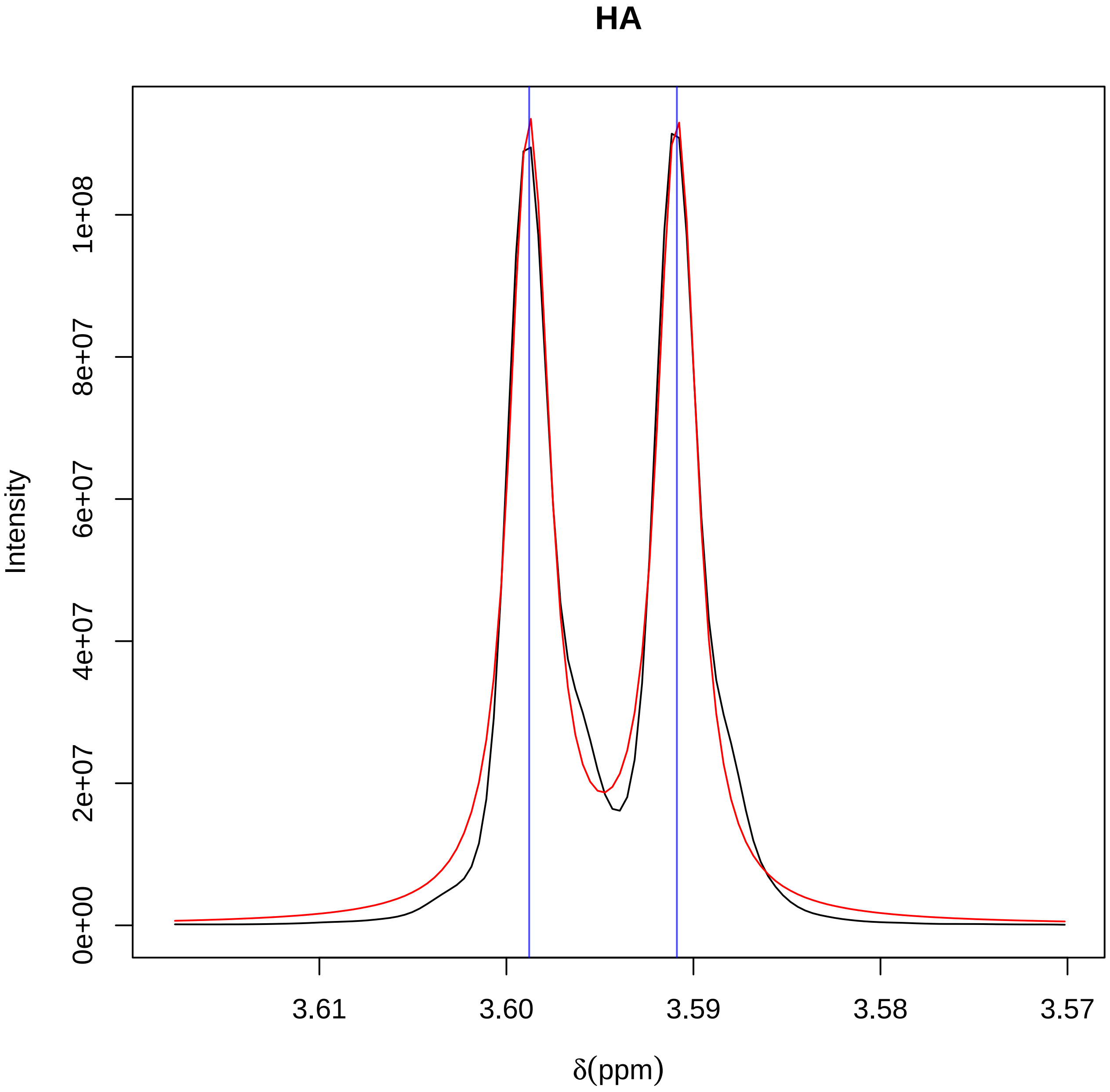

HA
Intensity
0e+00
2e+07
4e+07
6e+07
8e+07
1e+08
3.61
3.60
3.59
3.58
3.57
δ(ppm)

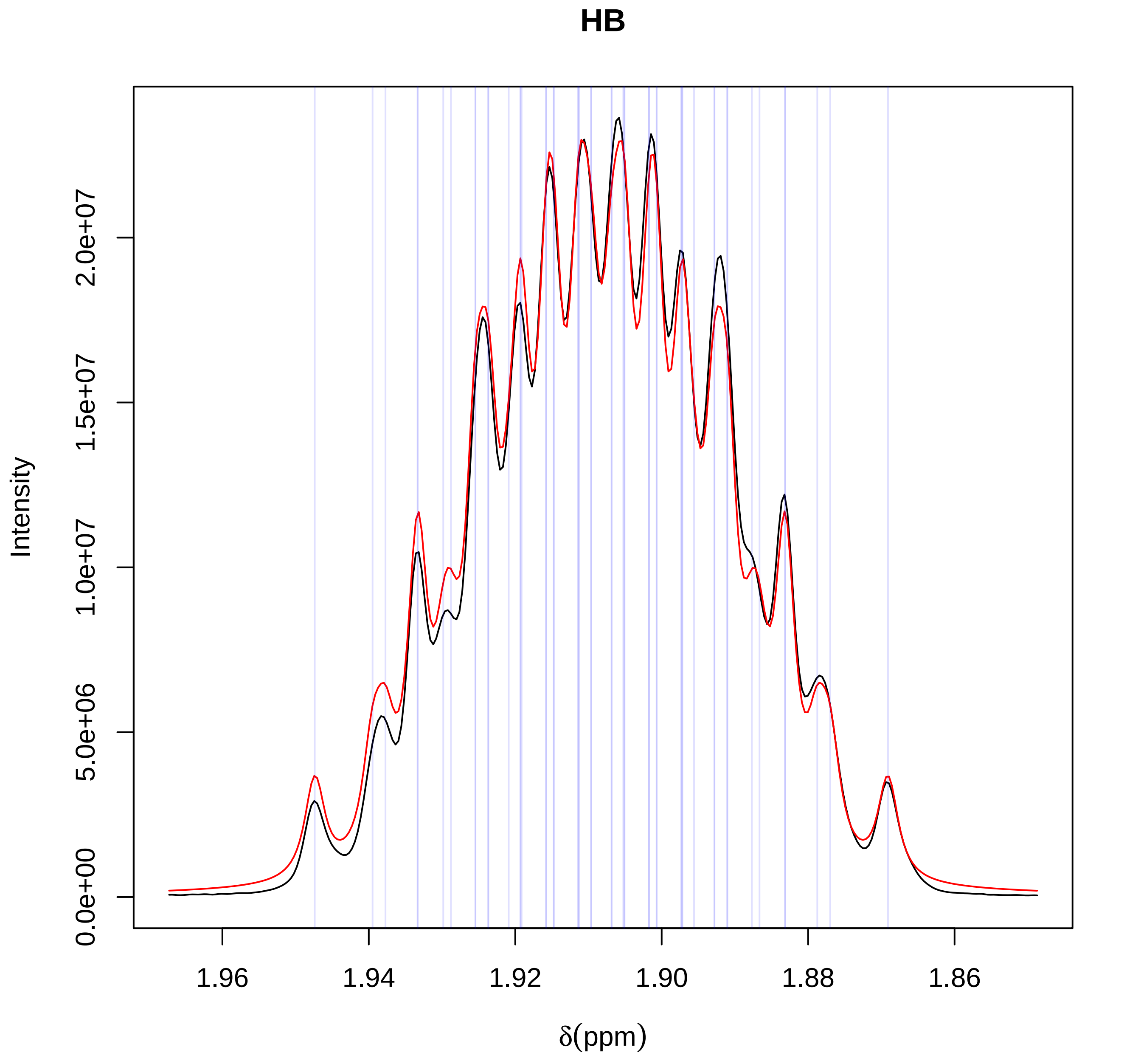
HB
Intensity
0.0e+00
5.0e+06
1.0e+07
1.5e+07
2.0e+07
1.96
1.94
1.92
1.90
1.88
1.86
δ(ppm)

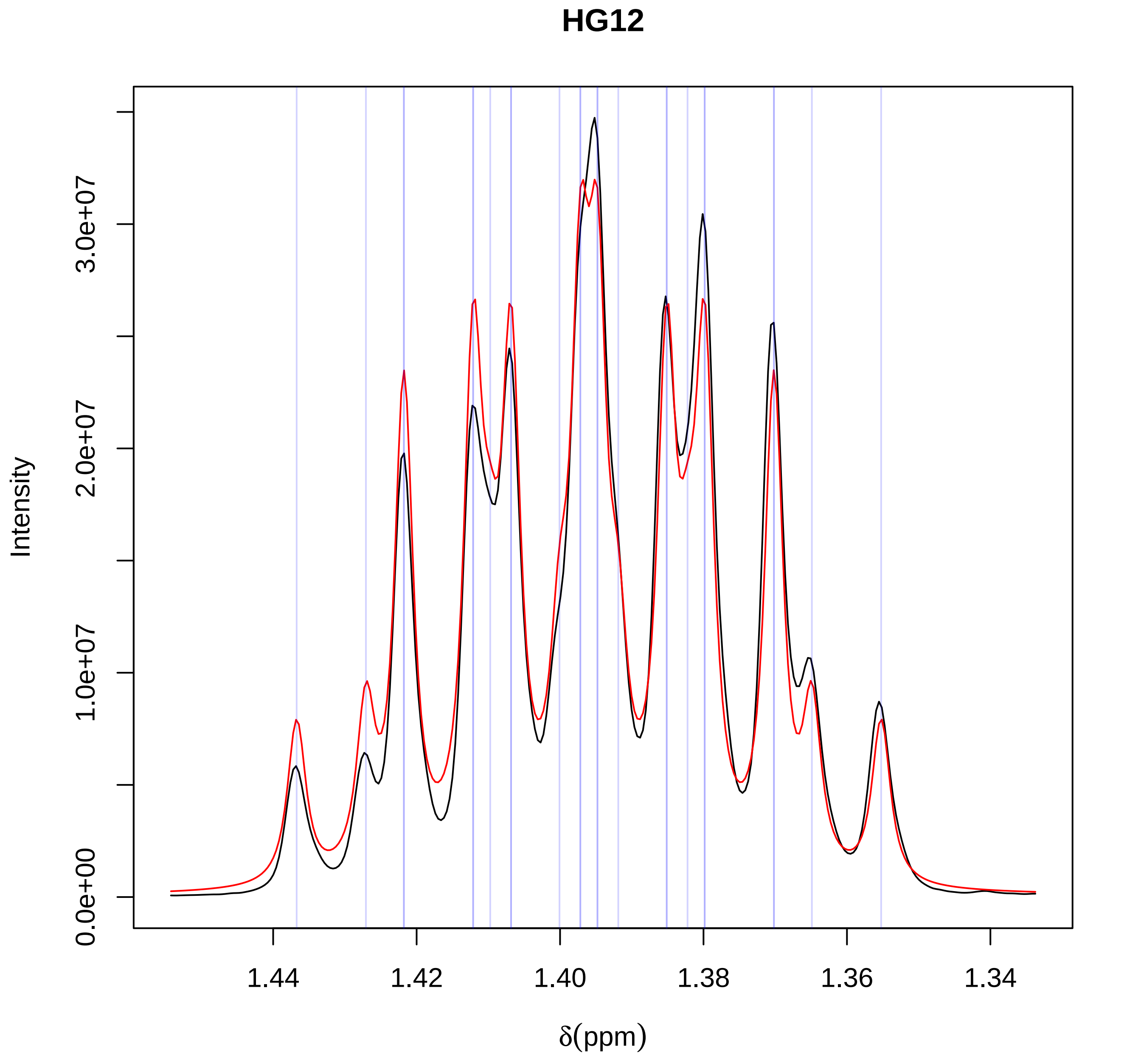

HG12
Intensity
0.0e+00
1.0e+07
2.0e+07
3.0e+07
1.44
1.42
1.40
1.38
1.36
1.34
δ(ppm)

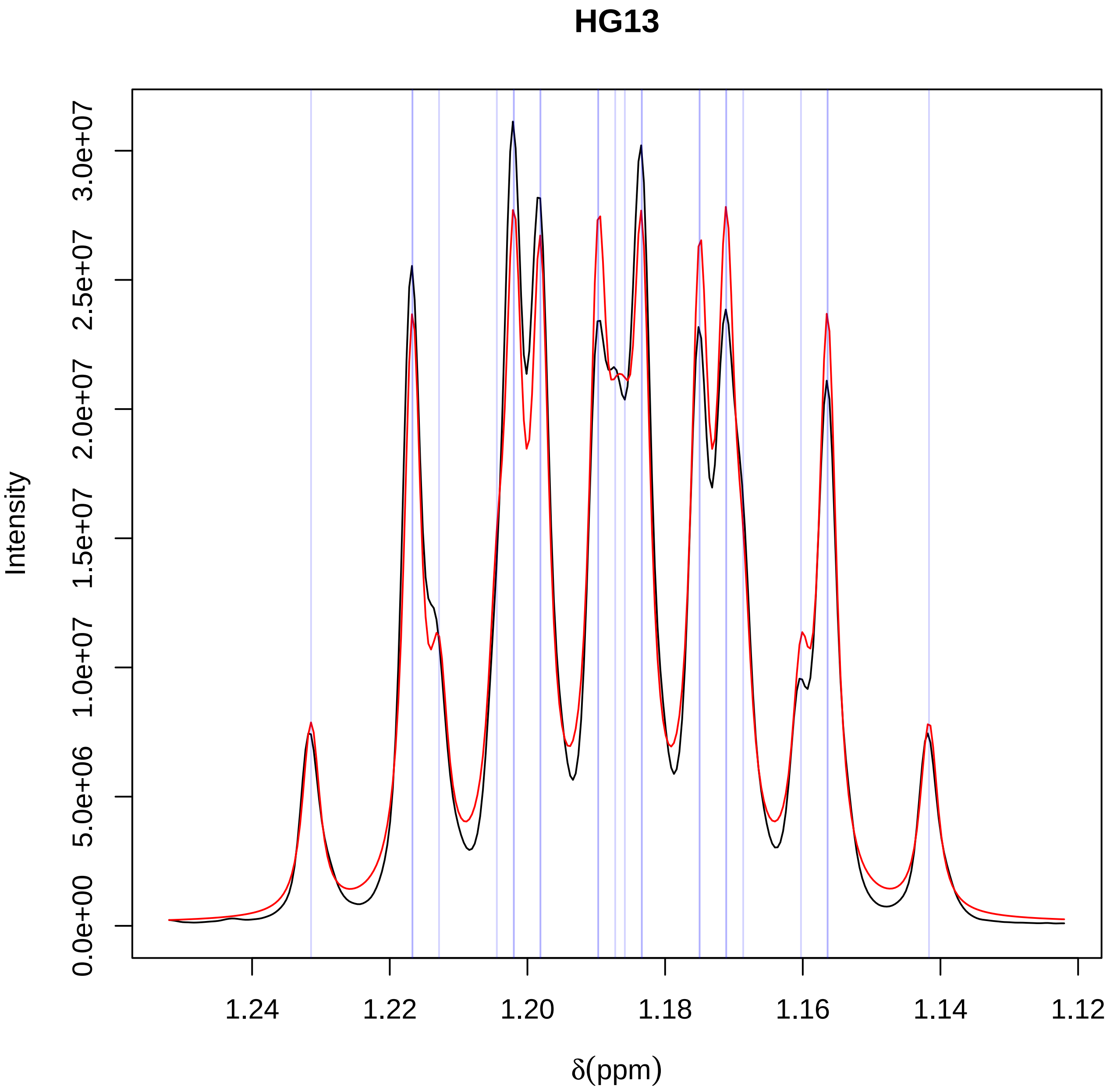
HG13
Intensity
0.0e+00
5.0e+06
1.0e+07
1.5e+07
2.0e+07
2.5e+07
3.0e+07
1.24
1.22
1.20
1.18
1.16
1.14
1.12
δ(ppm)

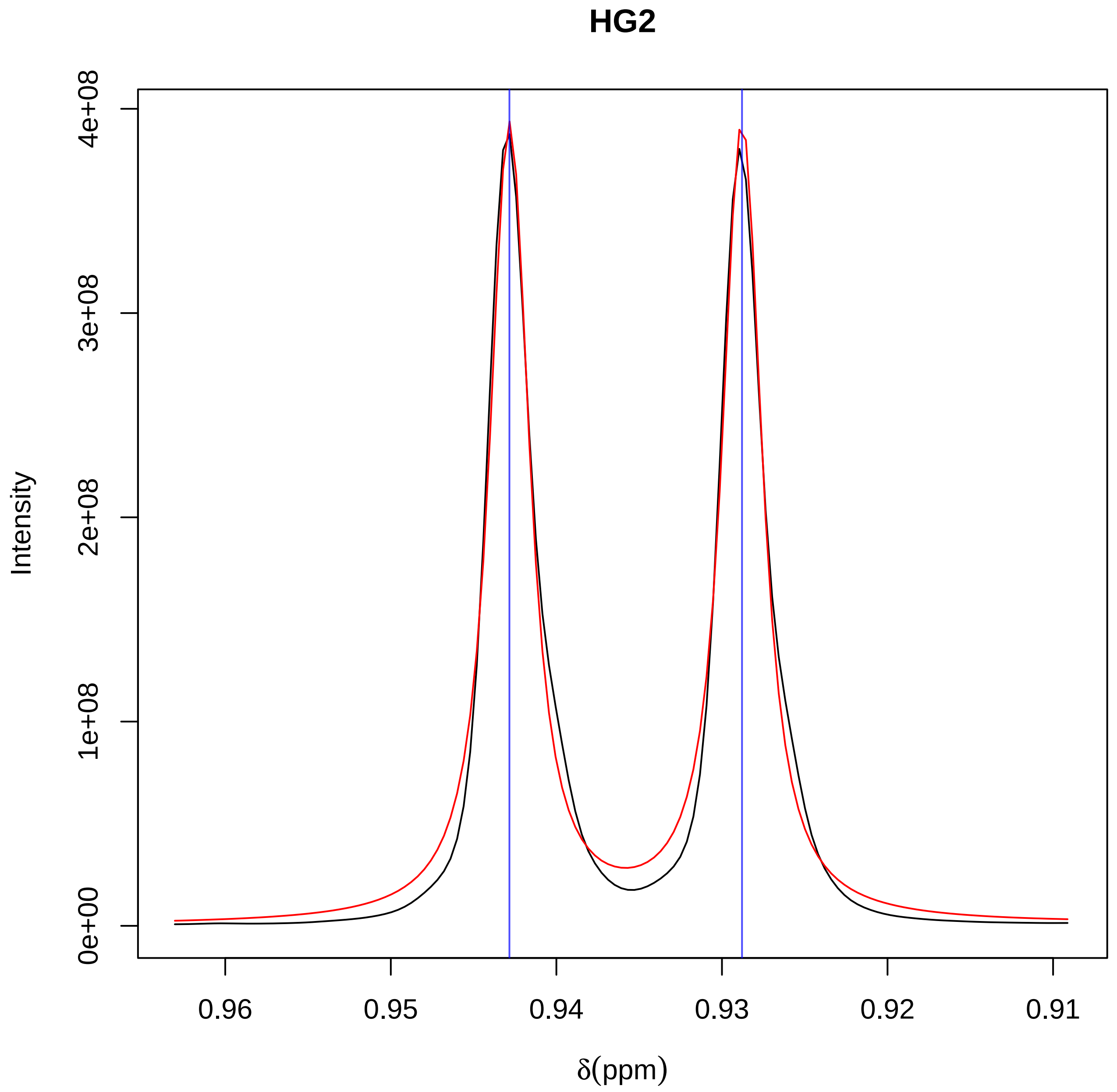

HG2
Intensity
0e+00
1e+08
2e+08
3e+08
4e+08
0.96
0.95
0.94
0.93
0.92
0.91
δ(ppm)

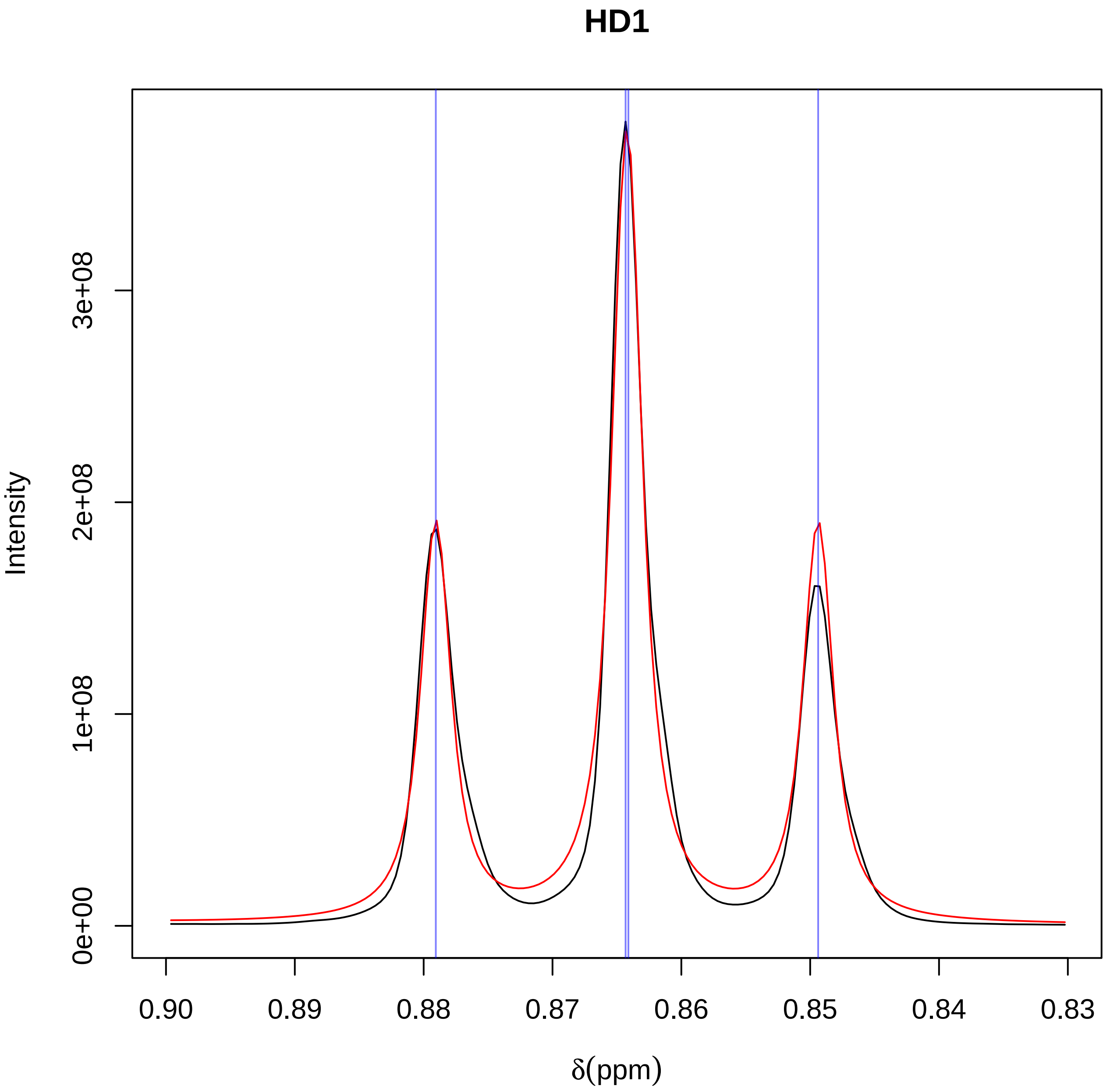

HD1
Intensity
0e+00
1e+08
2e+08
3e+08
0.90
0.89
0.88
0.87
0.86
0.85
0.84
0.83
δ(ppm)
